# Supplementary material for: COZOID: contact zone identifier for visual analysis of protein-protein interactions
Source: BMC Bioinformatics. 2018 Apr 6;19:125. doi: 10.1186/s12859-018-2113-6 (PMC5889581; doi:10.1186/s12859-018-2113-6)
Supplement: Supplementary file 2 — Software build. Executable binary file of the software tool. (ZIP 118,784 kb) [file 12859_2018_2113_MOESM2_ESM.zip › cozoid/java/docs/beansbinding-1.2.1-doc.zip!/org/jdesktop/beansbinding/package-use.html]

Uses of Package org.jdesktop.beansbinding (Beans Binding)


---


|  |  |  |
| --- | --- | --- |
| **Overview** \| **Package** \| Class \| **Use** \| **Tree** \| **Deprecated** \| **Index** \| **Help** | |  |
| PREV   NEXT | **FRAMES**    **NO FRAMES**     **All Classes** |  |


---


## **Uses of Package org.jdesktop.beansbinding**

| Packages that use org.jdesktop.beansbinding | |
| --- | --- |
| **org.jdesktop.beansbinding** | Provides support for defining properties and creating bindings between sets of two properties. |
| **org.jdesktop.swingbinding** | Provides support for binding to complex Swing components, and documentation on the interesting Swing properties to bind to. |
| **org.jdesktop.swingbinding.impl** | Private implementation details for Swing binding. |

Classes in org.jdesktop.beansbinding used by org.jdesktop.beansbinding

****AutoBinding****
  
          An implementation of `Binding` that automatically syncs the source
and target by refreshing and saving according to one of three update
strategies.

****AutoBinding.UpdateStrategy****
  
          An enumeration representing the possible update strategies of an
`AutoBinding`.

****BeanProperty****
  
          An implementation of `Property` that uses a simple dot-separated path
syntax to address Java Beans properties of source objects.

****Binding****
  
          `Binding` is an abstract class that represents the concept of a
binding between two properties, typically of two objects, and contains
methods for explicitly syncing the values of the two properties.

****Binding.SyncFailure****
  
          `SyncFailure` represents a failure to sync (`save` or `refresh`) a
`Binding`.

****Binding.SyncFailureType****
  
          An enumeration representing the reasons a sync (`save` or `refresh`)
can fail on a `Binding`.

****Binding.ValueResult****
  
          Encapsulates the result from calling
`Binding.getSourceValueForTarget()` or
`Binding.getTargetValueForSource()`, which
can either be a successful value or a failure.

****BindingListener****
  
          `BindingListeners` are registered on `Bindings` or `BindingGroups`
to listen for changes to the state of `Bindings`

****Converter****
  
          `Converter` is responsible for converting a value from one type
to another.

****ELProperty****
  
          An implementation of `Property` that allows Java Beans properties of
source objects to be addressed using a simple dot-separated path syntax
within an EL expression.

****ObjectProperty****
  
          An immutable, read-only, `Property` implementation whose `getValue`
method returns the source object that it is given.

****Property****
  
          `Property` defines a uniform way to access the value of a property.

****PropertyHelper****
  
          An abstract subclass of `Property` that helps with the management of
`PropertyStateListeners` by implementing the methods for adding, removing,
and getting listeners.

****PropertyStateEvent****
  
          An event characterizing a change in a `Property's` state for
a particular source object.

****PropertyStateListener****
  
          `PropertyStateListeners` are registerd on `Property`
instances, to be notified when the state of the property changes.

****Validator****
  
          `Validator` is responsible for validating the value from the target of
a `Binding`.

****Validator.Result****
  
          An instance of `Result` is returned from a `Validator's`
`validate` method to indicate an invalid value.

Classes in org.jdesktop.beansbinding used by org.jdesktop.swingbinding

****AutoBinding****
  
          An implementation of `Binding` that automatically syncs the source
and target by refreshing and saving according to one of three update
strategies.

****AutoBinding.UpdateStrategy****
  
          An enumeration representing the possible update strategies of an
`AutoBinding`.

****Binding****
  
          `Binding` is an abstract class that represents the concept of a
binding between two properties, typically of two objects, and contains
methods for explicitly syncing the values of the two properties.

****Property****
  
          `Property` defines a uniform way to access the value of a property.

Classes in org.jdesktop.beansbinding used by org.jdesktop.swingbinding.impl

****Binding****
  
          `Binding` is an abstract class that represents the concept of a
binding between two properties, typically of two objects, and contains
methods for explicitly syncing the values of the two properties.

****Property****
  
          `Property` defines a uniform way to access the value of a property.

---


|  |  |  |
| --- | --- | --- |
| **Overview** \| **Package** \| Class \| **Use** \| **Tree** \| **Deprecated** \| **Index** \| **Help** | |  |
| PREV   NEXT | **FRAMES**    **NO FRAMES**     **All Classes** |  |


---
